# The Impact of Emergency Physician Seniority on Clinical Efficiency, Emergency Department Resource Use, Patient Outcomes, and Disposition Accuracy

**DOI:** 10.1097/MD.0000000000002706

**Published:** 2016-02-12

**Authors:** Chao-Jui Li, Yuan-Jhen Syue, Tsung-Cheng Tsai, Kuan-Han Wu, Chien-Hung Lee, Yan-Ren Lin

**Affiliations:** From the Department of Emergency Medicine, Kaohsiung Chang Gung Memorial Hospital, Chang Gung University College of Medicine, Kaohsiung, Taiwan (C-JL, T-CT, K-HW); Department of Public Health, College of Health Science, Kaohsiung Medical University, Kaohsiung, Taiwan (C-JL, C-HL); Research Center for Environmental Medicine, Kaohsiung Medical University, Kaohsiung, Taiwan (C-HL); Department of Anesthesiology, Kaohsiung Chang Gung Memorial Hospital, Chang Gung University College of Medicine, Kaohsiung, Taiwan (Y-JS); Department of Emergency Medicine, Changhua Christian Hospital, Changhua, Taiwan (Y-RL); School of Medicine, Kaohsiung Medical University, Kaohsiung, Taiwan (Y-RL); and School of Medicine, Chung Shan Medical University, Taichung, Taiwan (Y-RL).

## Abstract

The ability of emergency physicians (EPs) to continue within the specialty has been called into question due to high stress in emergency departments (EDs).

The purpose of this study was to investigate the impact of EP seniority on clinical performance.

A retrospective, 1-year cohort study was conducted across 3 EDs in the largest health-care system in Taiwan. Participants included 44,383 adult nontrauma patients who presented to the EDs. Physicians were categorized as junior, intermediate, and senior EPs according to ≤5, 6 to 10, and >10 years of ED work experience. The door-to-order and door-to-disposition time were used to evaluate EP efficiency. Emergency department resource use indicators included diagnostic investigations of electrocardiography, plain film radiography, laboratory tests, and computed tomography scans. Discharge and mortality rates were used as patient outcomes. Disposition accuracy was evaluated by ED revisit rate.

Senior EPs were found to have longer door-to-order (11.3, 12.4 minutes) and door-to-disposition (2, 1.7 hours) time than nonsenior EPs in urgent and nonurgent patients (junior: 9.4, 10.2 minutes and 1.7, 1.5 hours; intermediate: 9.5, 10.7 minutes and 1.7, 1.5 hours). Senior EPs tended to order fewer electrocardiograms, radiographs, and computed tomography scans in nonurgent patients. Adjusting for age, sex, disease acuity, and medical setting, patients treated by junior and intermediate EPs had higher mortality in the ED (adjusted odd ratios, 1.5 and 1.6, respectively).

Compared with EPs with ≤10 years of work experience, senior EPs take more time for order prescription and patient disposition, use fewer diagnostic investigations, particularly for nonurgent patients, and are associated with a lower ED mortality rate.

## INTRODUCTION

It is generally believed that physicians who have more experience have also accumulated knowledge and skills during their years in practice, and are therefore able to deliver higher quality care. Clinical performance has been proven to be associated with physician experience and seniority.^1–4^ The literature indicates that junior doctors have incomplete doctor–patient communication skills,^5^ and have significantly greater levels of anxiety due to uncertainty.^6^

The ability of emergency physicians (EPs) to continue to practice their specialty for as long as specialists in other medical fields has been called into question due to high stress levels in emergency departments (EDs). Emergency physicians see a large volume of cases of varying complexity. The major concerns for EP clinical performance can be divided into 4 areas: the efficiency of patient assessment, the resource usage for patient diagnosis, the outcomes of treated patients, and the accuracy of disposition decisions. However, the relationship between clinical performance and the seniority of EPs is not well established, partly because emergency medicine is a relatively new medical specialty. The scarcity of senior EPs due to high levels of stress and burnout within the specialty also makes it more difficult to research differences in clinical performance between junior and senior EPs.^7^

The Chang Gung Memorial Hospitals constitute the largest health-care system in Taiwan. This medical complex includes 3 EDs in Northern Taiwan, with more than 300,000 cumulative mean annual ED visits. In these 3 EDs, there are 59 EPs whose total durations of employment in the ED ranging from 1 to 20 years. This large health-care system offered an opportunity to conduct a multicenter study in order to evaluate the differences in clinical performance between EPs. By assessing such data, a suitably thorough investigation could help to explain how disparities between EPs with different levels of seniority affect clinical practice. Based on this information, procedures could be developed to reduce differences in clinical practice, thereby helping to reduce variations in care, and potentially leading to better patient outcomes.

Given the possible influence of EP experience on diverse aspects of clinical performance, the present study investigated the hypotheses that the seniority of EPs would be associated with clinical efficiency, ED resource use, ED patient outcomes, and disposition accuracy. The purpose of this study was to evaluate the associations between the duration of EP experience and these aspects of clinical performance.

## MATERIALS AND METHODS

### Study Design

This retrospective, 1-year cohort study was approved by the Chang Gung Medical Foundation Institutional Review Board. Based on the understanding that all data regarding the patient and EP records that were used in the analyses had been anonymized and deidentified, the ethics committee approved the research protocol with a waiver of informed consent.

### Study Setting

This study was conducted across the Chang Gung Memorial Hospitals (the largest health-care system in Taiwan), which receive 8% to 10% of the National Health Insurance budget according to government statistics. From July 1, 2011 to June 30, 2012, 3 EDs within this health-care system were included in the study. Together, the 3 EDs account for more than 300,000 cumulative mean annual visits. One ED was in a tertiary referral medical center with over 3500 beds. The other 2 EDs were in secondary regional hospitals with over 1000 and 250 beds, respectively. The numbers of ED visits in the 2 larger hospitals are the greatest in their respective counties. In Taiwan, the National Health Insurance program covers more than 99% of the residents; thus, ED medical service is accessible to almost all individuals. The patient is only responsible for a part of the medical expenses, which depends on the degree of the medical setting (approximately, $23 and $18 US dollars for the tertiary referral medical center and secondary regional hospital, respectively). This medical expense covered all of the routine examinations that were used in this investigation.

### Patients

All adult nontrauma patients who presented to the EDs during the day shift were considered for inclusion in the patient sample. To evaluate the clinical performance of EPs for an entire year, all ED patients in a defined year were included in this study. The day shift was 8 hours (7:00–15:00), based on the EPs’ duty hours. Because the 3 studied EDs were all teaching medical units, residents assisted with the treatment of ED patients under the supervision of EPs. To focus this investigation on the clinical performance of EPs, patients treated by residents were excluded from the data analysis. Door-to-order time, door-to-disposition time, the use of computed tomography (CT) scans, and mortality rates were used to represent the EP clinical performance variables for the power calculation. Given a 2-tailed test with a type I error of 0.05, the statistical power for the analyses of the study patients (n = 44,383) was calculated to be more than 93.5% in a post hoc analysis.

### Emergency Physicians

In total, the study included 59 full-time EPs with seniority ranging from 1 to 20 years. These EPs rotated throughout the 3 EDs and were categorized into 3 groups according to seniority: junior (n = 26; ≤5 years of work experience), intermediate (n = 12; 6–10 years of work experience), and senior (n = 21; >10 years of work experience). The 59 EPs received the same residency training program, which had been developed by the Taiwan Society of Emergency Medicine. In the tertiary referral medical center, 3 attending EPs worked together in the same day shift and the patients were assigned to different attending physicians according to the ED triage. One physician was in charge of first- and second-level triage patients, 1 physician was in charge of third-level triage patients, and the other physician was in charge of fourth- and fifth-level triage patients. In one of the secondary regional hospitals, 2 attending EPs worked together in the same day shift and the patients were assigned alternately to each attending EP. In the other secondary regional hospital, there was only 1 attending EP per day shift who was in charge of the medical work.

### Study Protocol

The demographic and clinical factors (age, sex, clinical urgency, and ED length of stay) of the patients were drawn from the ED administrative database and studied in reference to the seniority of the attending EP. Clinical urgency was defined according to the five-level Taiwan triage and acuity scale, formulated by the Department of Health in Taiwan. According to these criteria, patients identified as triage levels 1 and 2 should be seen immediately or within 10 minutes, respectively, and are defined as urgent. Patients with triage levels 3, 4, and 5 should be assessed within 30, 60, or 120 minutes, respectively, and are classified as nonurgent. The assessment of clinical performance was divided into 4 areas: EP efficiency, ED resource use, patient outcomes, and the accuracy of disposition decision.

### Measures

To evaluate EP efficiency, the time interval between patient registration and the prescription of the first order by the EP (door-to-order time) was recorded, as was the time interval between patient registration and the completion of patient disposition by the EP (door-to-disposition time). To evaluate ED resource use, the diagnostic investigations ordered by the EPs were documented. Investigations included electrocardiography (EKG), plain film radiography, laboratory tests (such as complete blood count, blood chemistry, urine analysis, stool analysis, or influenza screen test), and CT scans. The discharge and mortality rates were used to represent patient outcomes in order to evaluate quality of care. Discharge rate was defined as the number of patients discharged home after being attended to in the ED divided by the total number of ED patients. The mortality rate was defined as the number of deaths within the ED divided by the total number of ED patients. To assess the accuracy of disposition, data were collected on 72-hour ED revisits (ie, the patients who returned to the ED within the 72 hours after discharge). The 72-hour revisit rate was defined as the number of revisit patients within 72 hours divided by the total number of ED patients. Data on EP clinical efficiency, clinical resource use, patient outcomes, and patient disposition were measured using ED electronic systems.

### Data Analysis

For continuous variables, the data are summarized as the mean and standard deviation. Because the distributions of the door-to-order time and the door-to-disposition time were not normal, medians with interquartile ranges, and nonparametric Kruskal–Wallis tests were used to describe and evaluate the associations with EP seniority. The distributions of categorical demographic factors (sex, disease acuity, and medical setting), clinical variables (the use of EKG, plain film radiography, laboratory tests, and CT), patient outcomes (discharge and ED mortality), and disposition accuracy (ED revisit in 72 hours) were summarized as numbers and percentages, as analyzed according to EP seniority. χ^2^ tests were used to evaluate the associations between these parameters and EP seniority, and χ^2^ tests for trend were employed to examine dose-response relationships. In the multivariate analyses, binary logistic regression models were applied to assess the effects of EP seniority on ED resource use, patient outcomes, and disposition accuracy. Effects were estimated in terms of odds ratios and the corresponding 95% confidence intervals (CIs) after adjusting for age, sex, disease acuity, and medical setting. Results were considered statistically significant for a 2-tailed *P* < 0.05. The statistical analysis was conducted using SPSS version 12.0 (SPSS, Chicago, IL) for Windows.

## RESULTS

### Patient Characteristics

During the 1-year study, 68,282 adult nontrauma patients visited the 3 EDs during the day shifts. Of this patient sample, 23,899 patients who were treated by residents under EP supervision were excluded, and the remaining 44,383 patients who were only treated by EPs were included in the final study cohort. Junior, intermediate, and senior EPs separately evaluated 19,759 (44.5%), 10,146 (22.9%), and 14,478 (32.6%) patients in the study cohort. The mean number of patients seen per hour in a shift was 2.3, 2.4, and 2.4 for junior, intermediate, and senior EPs, respectively (*P* = 0.202). The patients’ basic demographic factors are shown in Table 1. Junior and intermediate EPs tended to treat older patients (age, 55.4 and 55.8 years, respectively), more male patients (51.3% and 55.5%, respectively), more urgent patients (11.4 and 11.7%, respectively) than did senior EPs (54.6 years, 49.9%, and 9.3%, respectively). In contrast, a higher proportion of senior EPs worked in the medical center (79.8%) than did junior and intermediate EPs (62.9% and 66.6%, respectively).

**TABLE 1:**
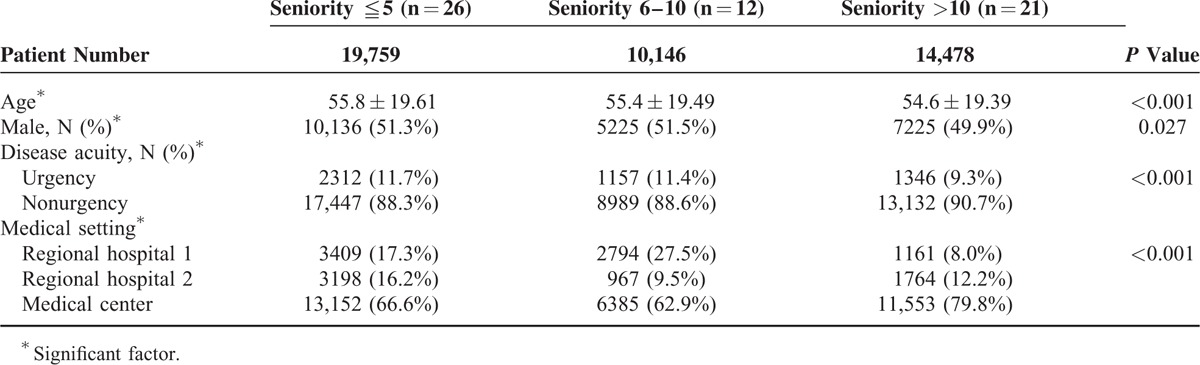
Demographic Factors of Patients in the Emergency Departments, Stratified by the Seniority of Emergency Physicians

### Clinical Performance Associated With Seniority

**FIGURE 1:**
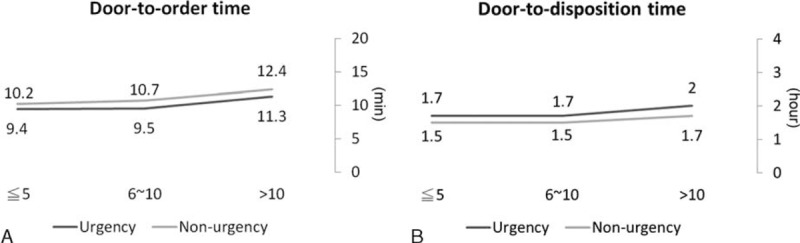
The distribution of door-to-order time (A) and door-to-disposition time (B) clinically made by the emergency physicians among urgent and nonurgent patients. Note: door-to-order time (minutes) represents the time interval between patient registration and emergency physician prescribing the first order, and door-to-disposition time (hours) represents the time interval between patient registration and emergency physician completing disposition order. The 2 types of data were presented as median with interquartile ranges and nonparametric Kruskal–Wallis tests were used to evaluate the differences. Both *P* < 0.001 for door-to-order time and door-to-disposition time. (Door-to-order time, urgent: ≤5: 9.4 [5.6], 6–10: 9.5 [6.06], >10: 11.3 [7.33]; nonurgent: ≤5: 10.2 [7.32], 6–10: 10.7 [7.83], >10: 12.4 [9.69]; door-to-disposition time, urgent: ≤5: 1.7 [1.27], 6–10: 1.7 [1.23], >10: 2.0 [1.69]; nonurgent: ≤5: 1.5 [1.46], 6–10: 1.5 [1.45], >10: 1.7 [1.74]; data are median [interquartile range)]).

**FIGURE 2:**
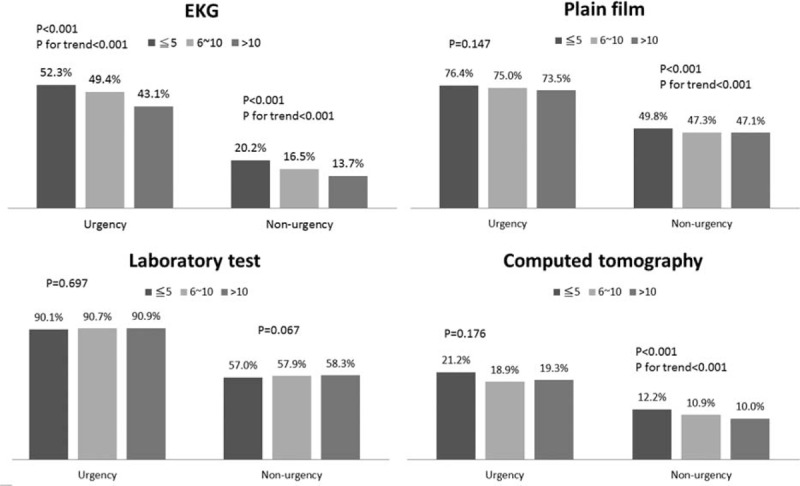
The distribution of emergency department resource use associated with the seniority of emergency physicians among urgent and nonurgent patients.

**FIGURE 3:**
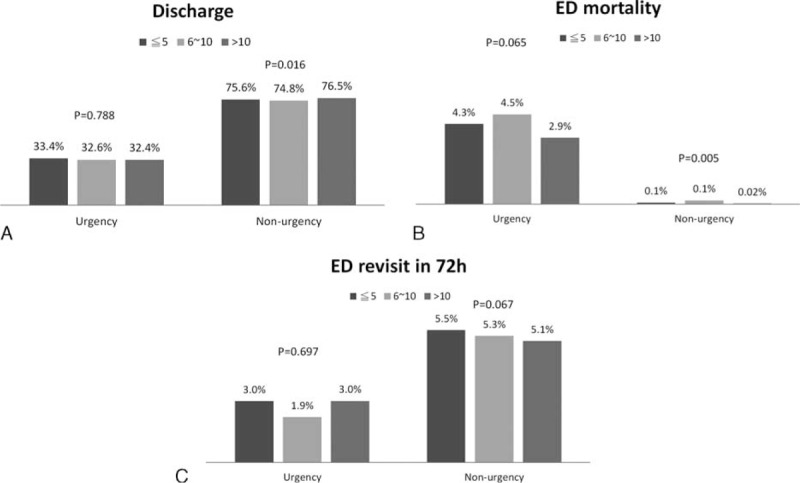
The distribution of patient outcomes (patient discharge rate, [A]; emergency department mortality, [B], and emergency department revisit rate in 72 hours, [C]) associated with the seniority of emergency physicians among urgent and nonurgent patients.

As shown in Figure 1, there were significant associations between EP seniority and EP efficiency in urgent and nonurgent patients (*P* < 0.001 for both door-to-order time and door-to-disposition time, Kruskal–Wallis test). Emergency physicians with >10 years of work experience were found to have a longer door-to-order and door-to-disposition time than did EPs with ≤10 years of work experience (all *P* < 0.001). Emergency department resource use was also associated with seniority (Figure 2). Senior EPs tended to order fewer EKGs, plain film examinations, and CT scans than did nonsenior EPs. A significantly decreased trend in the EKG use rate was found for urgent and nonurgent patients, as well as for plain film and CT scans in nonurgent patients (all *P* for trend <0.05). The laboratory tests ordered by the EPs were not significantly associated with seniority. In contrast, specific patient outcomes were found to be associated with the seniority (Figure 3). Among nonurgent patients, senior EPs showed a higher patient discharge rate (76.5%) and a lower ED mortality rate (0.02%) than did the other 2 groups of EPs (74.8% and 75.6% and 0.1%, respectively). The ED revisit rate in 72 hours was not significantly different across the 3 EPs groups.

After adjusting for age, sex, disease acuity, and medical setting, the junior and intermediate EPs had a higher likelihood of ordering an EKG (adjusted odds ratio [aOR] = 1.6; 95% CI: 1.46–1.64 and aOR = 1.2, 95% CI: 1.12–1.29, respectively) and CT (aOR = 1.3, 95% CI: 1.22–1.40 and aOR = 1.2, 95% CI: 1.07–1.26, respectively) than did senior EPs (Table 2). As compared with the senior EPs, the junior EPs were also found to order more plain films (aOR = 1.1, CI: 1.07–1.18). The associations of EP seniority with patient outcomes and disposition accuracy are shown in Table 3. After adjusting for the confounding factors that are mentioned above, the junior and intermediate EPs showed higher patient ED mortality rates than did the senior EPs (aOR = 1.5, 95% CI: 1.02–2.20 and aOR = 1.6, 95% CI: 1.04–2.43, respectively). Data from the multivariate analysis showed that patient discharge and ED revisit in 72 hours were not significantly related to the seniority of EPs.

**TABLE 2:**
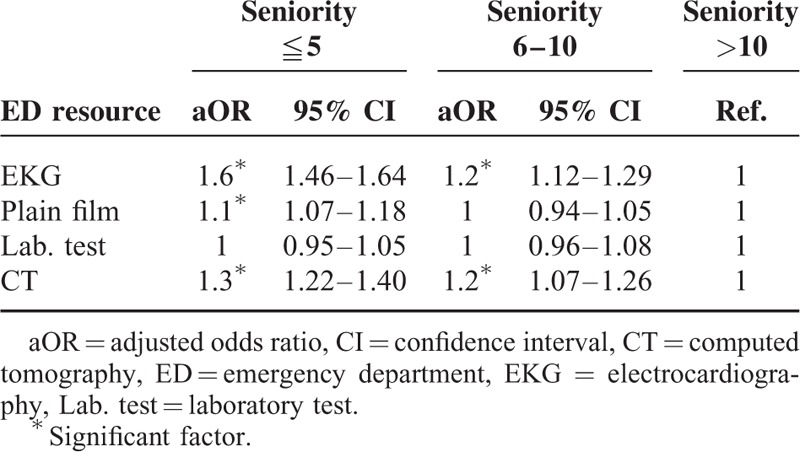
The Association Between Emergency Physician Seniority and Emergency Department Resource Use by Logistic Regression, Adjust for the Age, Sex, Disease Acuity, and Medical Setting

**TABLE 3:**
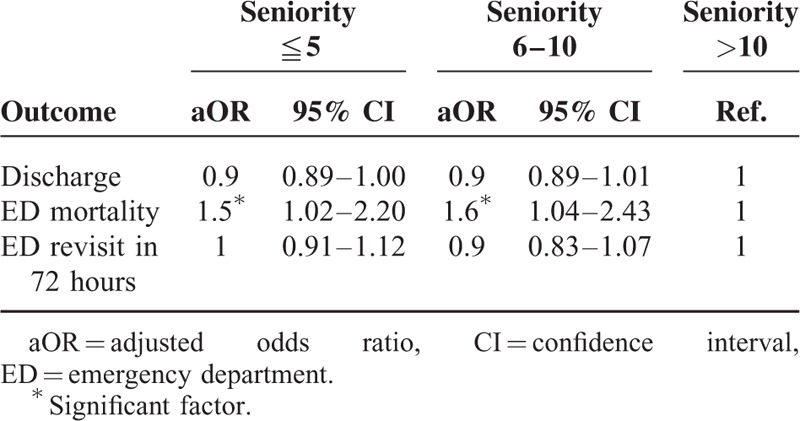
The Association Between Emergency Physician Seniority and Patient Outcome by Logistic Regression, Adjust for the Age, Sex, Disease Acuity, and Medical Setting

## DISCUSSION

Earlier studies have reported that senior doctor input in ED patient care added accuracy to disposition decisions, assisted with patient safety, and improved departmental flow.^8,9^ The present study arrived at similar findings in that the clinical performance of senior EPs was found to be better than that of their junior colleagues. Although senior EPs took more time to order prescriptions and make disposition decisions for ED patients, the measures related to ED resource usage and patient outcomes were better than those observed for nonsenior EPs. However, the findings of the present study differ from those reported by Niteesh et al, in which senior physicians were observed to be less likely to deliver high-quality care.^4^ A portion of the discrepancy between these results may be explained by the different study groups and outcome measures. The previous study investigated primary care and family medicine physician groups, and the evaluations were focused on the relationship between physician's age and adherence to standards of appropriate diagnosis, screening, preventive health care, and appropriate therapy. In contrast, the research focus of the present study was the associations between the ED experience of EPs and their clinical performance and patient outcomes.

The present study's findings regarding the additional time that senior EPs took to order prescriptions and make disposition decisions are compatible with those reported by Wu et al.^10^ In Wu et al's study, senior EPs were found to keep patients under observation in the ED for longer periods than their junior colleagues. It has been recognized that senior EPs are more aware of the fact that “medicine is an uncertain science,” as compared with junior EPs. Therefore, senior EPs evaluate patients repeatedly. For example, an EP cannot predict whether a patient with a head injury will go on to develop a delayed intracranial hemorrhage based on an initial CT scan. Accordingly, a senior EP might allow more time to observe clinical changes, repeat neurologic examinations, and instruct the patient regarding how he or she could monitor focal neurologic deficits by himself or herself. This might explain the longer times that senior EPs generally took to make disposition decisions for ED patients.

A previous investigation that compared ED performance between residents and physicians showed that physician experience and the severity of patient illness were both associated with the volumes of tests that were performed in the EDs of teaching hospitals.^8^ The present study also demonstrated that greater EP seniority was related to a decrease in the proportion cases in which plain film radiography or CT scans were ordered; however, a significant trend was only found among nonurgent ED patients (and was not observed among urgent ED patients). Given that the senior EPs were observed to offer better medical care (as reflected by lower ED patient mortality), greater EP experience appeared to be an important factor for better use of radiography, and CT scans among nonurgent ED patients. Similar results were also found for the use of EKG among urgent and nonurgent ED patients. These results imply that the transmission of medical experience should be stressed in regarding the use of these types of resources in the ED, which could help to save medical resources and reduce unnecessary radiation exposure.

This investigation demonstrated that nontrauma ED patients who were clinically treated by the senior EPs had a significantly lower mortality rate (0.02%) than did the corresponding patients who were clinically treated by nonsenior EPs (0.1%). This finding resembles the result reported by Wyatt et al,^11^ who observed that major trauma patients had improved outcomes when they were treated by a senior physician. The rates of home discharge, patient mortality, and emergency revisits in 72 hours were measurable aspects of the quality of patient care. Adjusting for patient age, sex, clinical urgency, and medical setting, the present study revealed that junior and intermediate EPs were associated with 1.5- to 1.6-fold higher ED patient mortality. This finding indicates that the lower ED patient mortality that was observed among patients treated by the senior EPs might have been related to the clinical experience of these EPs.

The most commonly addressed stressors in modern medicine include long and irregular work hours, low financial remuneration, and demands of family life.^12^ The practice of emergency medicine has been identified as a field with high risks for medical error.^13–15^ The ability of EPs to continue to practice their specialty for as long as the specialists in other medical fields (ie, over an entire career) has been called into question due to the high levels of stress that this type of medical practice generates.^7,12,16,17^ The results of this study demonstrate that, as compared with their junior colleagues, senior EPs could continue to offer a significant clinical workload with better clinical performance.

## LIMITATIONS

This study has several limitations. First, the 3 study hospitals belonged to the same health-care system, which might limit the generalizability of the conclusions. Second, data were not available in the electronic system with regard to technical quality and the appropriateness of clinical care, which limits the evaluation of the effect of medical care factors on patient outcomes. Finally, only a limited collection of confounding factors was taken into consideration; therefore, the results of this study should be interpreted in a cautious manner.

## CONCLUSIONS

In summary, as compared with EP colleagues who have ≤10 years of work experience, senior EPs take more time to order prescriptions and make disposition decisions. Additionally, these senior EPs use fewer diagnostic procedures, particularly for nonurgent patients. Finally, the greater experience of these senior EPs is associated with a lower ED mortality rate. The findings of this study emphasize that the clinical experience derived from a career is important to EPs.

## References

[1] Sacchetti A Carraccio C Harris RH Resident management of emergency department patients: is closer attending supervision needed. *Ann Emerg Med* 1992; 21:749–752. 1590624 10.1016/s0196-0644(05)82797-0

[2] Haas J Orav EJ Goldman L The relationship between physicians qualifications and experience and the adequacy of prenatal care and low birthweight. *Am J Public Health* 1995; 85:1087–1091. 7625501 10.2105/ajph.85.8_pt_1.1087 PMC1615802

[3] Vicente G Lomar FP Mlot C Can the experienced ICU physician predict ICU length of stay and outcome better than less experienced colleagues. *Intensive Care Med* 2004; 30:655–659. 14735235 10.1007/s00134-003-2139-7

[4] Choudhry NK Fletcher RH Soumerai SB Systemic review: the relationship between clinical experience and quality of health care. *Ann Intern Med* 2005; 142:260–273. 15710959 10.7326/0003-4819-142-4-200502150-00008

[5] Canwell BM Rarnirez AJ Doctor-patient communication: a study of junior house officers. *Med Educ* 1997; 31:17–21. 10.1111/j.1365-2923.1997.tb00037.x 9231119

[6] Bovier PA Perneger TV Stress from uncertainty from graduation to retirement: a population-based study of Swiss physicians. *J Gen Intern Med* 2007; 22:632–638. 17443371 10.1007/s11606-007-0159-7 PMC1855273

[7] Doan-Wiggins L Zun L Cooper MA Practice satisfaction, occupational stress, and attrition of emergency physicians. *Acad Emerg Med* 1995; 2:556–563. 7497060 10.1111/j.1553-2712.1995.tb03261.x

[8] Salazar A Corbella X Onaga H Impact of a resident strike on emergency department quality indicators at an urban teaching hospital. *Acad Emerg Med* 2001; 8:804–808. 11483455 10.1111/j.1553-2712.2001.tb00210.x

[9] White AL Armstrong PAR Thakore S Impact of senior clinical review on patient disposition from the emergency department. *Emerg Med J* 2010; 27:262–265. 20385673 10.1136/emj.2009.077842

[10] Wu K-H Chen I-C Li C-J The influence of physician seniority on disparities of admit/discharge decision making for ED patients. *Am J Emerg Med* 2012; 30:1555–1560. 22424989 10.1016/j.ajem.2012.01.011

[11] Wyatt JP Henry J Beard D The association between seniority of accident and emergency doctor and outcome following trauma. *Injury* 1999; 30:165–168. 10476260 10.1016/s0020-1383(98)00252-6

[12] Hall KN Wakeman MA Richard C Factors associated with career longevity in residency-trained emergency physicians. *Ann Emerg Med* 1992; 21:291–297. 1536490 10.1016/s0196-0644(05)80890-x

[13] Brennan TA Leape LL Laird NM Incidence of adverse events and negligence in hospitalized patients: results of the Harvard Medical Practice Study I. *N Engl J Med* 1991; 324:370–376. 1987460 10.1056/NEJM199102073240604

[14] Keller KL Koenig WJ Management of stress and prevention of burnout in emergency physician. *Ann Emerg Med* 1989; 18:42–47. 2783361 10.1016/s0196-0644(89)80309-9

[15] Vincent C Simon R Sutcliffe K Errors conference: executive summary. *Acad Emerg Med* 2000; 7:1180–1182. 11073464 10.1111/j.1553-2712.2000.tb00461.x

[16] Hall KN Wakeman MA Residency-trained emergency physicians: their demographics, practice evolution, and attrition from emergency medicine. *J Emerg Med* 1999; 17:7–15. 9950379 10.1016/s0736-4679(98)00119-x

[17] Ros SP Scheper R Career longevity in clinical pediatric emergency medicine. *Pediatr Emerg Care* 2009; 25:487–488. 19633591 10.1097/PEC.0b013e3181b09d19

